# Case report: Sudden unexpected death due to tuberculous myocarditis involving sinus node at autopsy

**DOI:** 10.3389/fcvm.2023.1159292

**Published:** 2023-06-15

**Authors:** Le Zhang, He Yan, Yufang Wang, Feijun Huang

**Affiliations:** ^1^ Forensic Science Center, Gannan Medical University, Ganzhou, China; ^2^ Department of Forensic Science, School of Basic Medical Sciences, Central South University, Changsha, China; ^3^ Department of Forensic Science, West China School of Basic Medical Sciences & Forensic Medicine, Sichuan University, Chengdu, China

**Keywords:** tuberculous myocarditis, sinus node, *Mycobacterium tuberculosis*, pulmonary tuberculosis, myocardial tuberculosis

## Abstract

Tuberculous myocarditis (TM) is an extremely rare manifestation of *Mycobacterium tuberculosis* (TB) infection. Although TM is a critical cause of sudden cardiac death, only a few cases have been reported. We report the case of an older patient with pulmonary TB with a history of fever, chest tightness, paroxysmal palpitations, and electrocardiographic evidence of sinus node conduction abnormalities on admission. Although emergency physicians observed these unusual clinical manifestations, no timely differential diagnosis was made nor interventions were performed. A definitive diagnosis of TM and histopathological findings compatible with sinus node involvement were made based on autopsy outcomes. Herein, we describe the clinical presentation and pathological features of a rare form of *Mycobacterium TB*. In addition, we provide an overview of issues related to the diagnosis of myocardial TB.

## Introduction

Although tuberculosis (TB) is a curable disease, it is one of the top 10 causes of death and the leading cause of mortality from a single infectious disease globally (ranking above human immunodeficiency virus and malaria) (1). Therefore, tubercle bacilli infection remains a significant global public health issue (2). The World Health Organization estimates that approximately 10 million new cases of TB and 1.3 million TB-related deaths occur annually (3). More than 95% of deaths due to TB occur in developing and less developed countries (4). In addition, the global risk of TB-associated infection and mortality has increased significantly in recent years owing to the substantial destruction of the public health system due to the recent COVID-19 pandemic (5, 6).

TB is a multisystemic disease that can infect any body organ; however, specific body sites, such as the heart, thyroid, and skeletal muscles, are rarely affected (7). Tuberculous myocarditis (TM) is a rare type of extrapulmonary TB. Only 1%–2% of all patients with TB have heart involvement, and <0.3% die due to the disease (8, 9). Nonetheless, the literature on myocardial TB is limited. The first case of TM was reported in 1664, and sudden death due to myocardial TB was first described in 1977 (7, 10). The clinical symptoms of myocardial TB are diverse and lack specificity, and TB infection often coexists in other organs. To this end, it can be challenging to obtain a timely and accurate judgment in the early diagnosis of TB in clinical practice; TB is usually diagnosed by postmortem examination (11).

Due to the lack of large-scale clinical samples and data relating to myocardial TB, the reporting and dissemination of such cases is very significant. Here, we describe a unique case of sudden unexpected death due to TM in a patient with the indication of sinus node conduction abnormalities as the initial presentation. A definitive diagnosis was established based on postmortem autopsy findings and pathological examinations. This case report describes in detail the clinical manifestations, pathological features, physical examinations, and laboratory tests outcomes of the patient to raise awareness about TM.

## Case description

A 55-year-old male patient was admitted to the hospital with complaints of fever, cough, chest tightness, and paroxysmal palpitations for 1 week. He had a history of TB infection of the lungs 2 years ago. He received routine first-line anti-TB therapy for over 8 months (including isoniazid, rifampin, ethambutol, and pyrazinamide) and was discharged.

**Figure 1:**
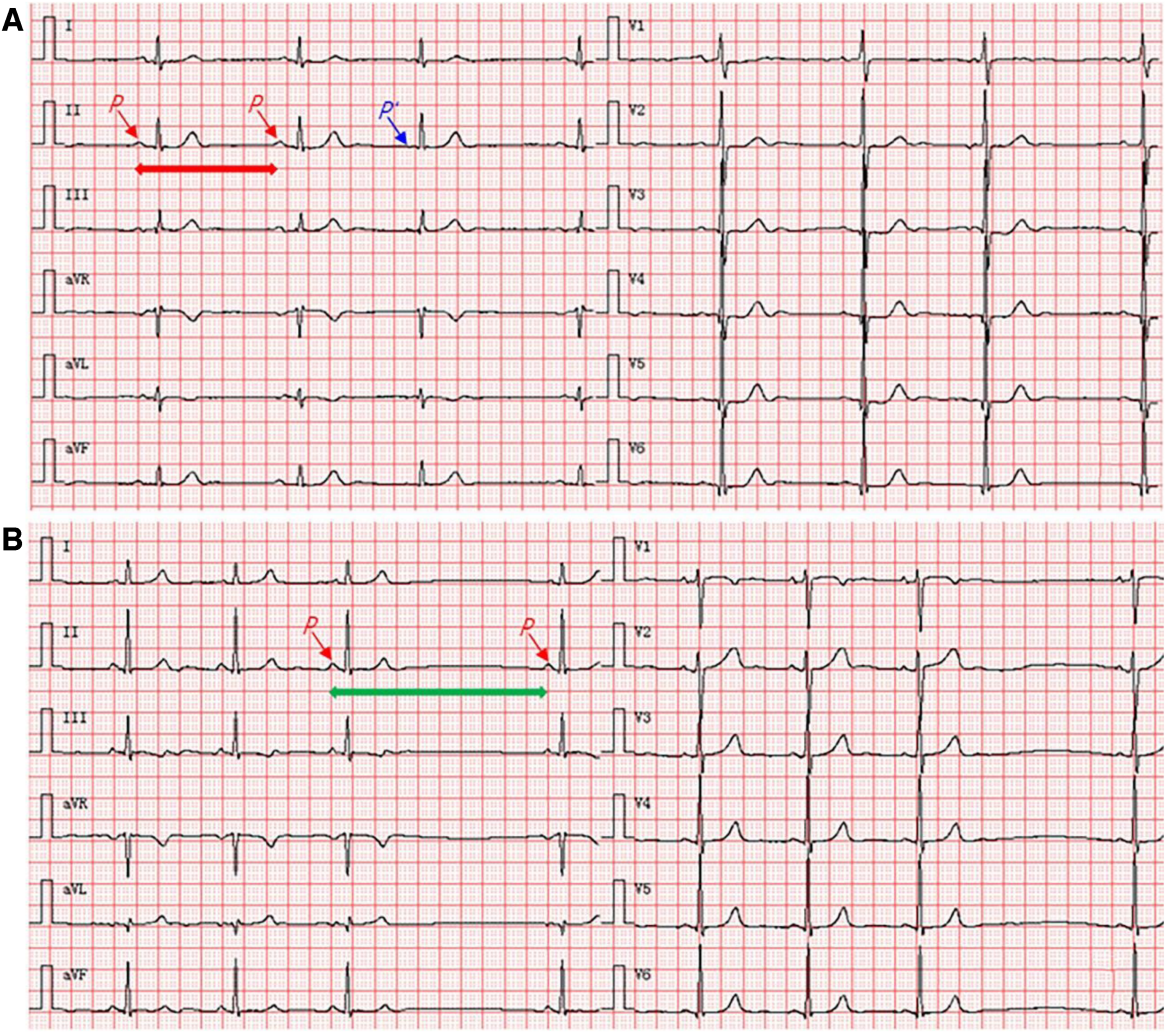
Twelve-lead synchronous ECG shows sinus node conduction abnormalities. (**A**) An increase in the *P*–*P* intervals and the rhythm of <60 beats/min represent sinus bradycardia (take longer horizontal red arrow blue in the lead II as the example); the third *P*-wave (*P*′) in all 12 leads that occurred earlier indicates premature atrial beats (take shorter horizontal blue arrow in the lead II as the example). (**B**) A single missed beat occurred between the third and fourth *P*-waves, but the increase in *P*–*P* interval is twice as long as the previous *P–P* intervals in all 12 leads (take longer horizontal green arrow in the lead II as the example), indicating the occurrence of type II second-degree sinoatrial block. ECG, electrocardiogram.

At admission, the patient has discontinued anti-TB treatment and was not taking any medication, including hormone preparations, to control other chronic diseases. Additionally, he denied a history of diabetes, hypertension, or tumors. Notably, he reported several sudden chest pain episodes lasting for a few seconds, accompanied by dizziness and amaurosis during the past week. He underwent a general clinical examination at a local outpatient community hospital setting. Previous medical records showed temporary sinus bradycardia with premature atrial beats on electrocardiogram (ECG) (Figure 1A), which spontaneously recovered to a normal rhythm after rest.

Based on physical examination at admission, he had a height of 169 cm, body mass index of 18.13 kg/m^2^, temperature of 36.9°C, respiration rate of 18 breaths/min, blood pressure of 109/64 mmHg, and mild malnutrition. No anemia or palpable superficial node enlargement were detected. ECG showed a type II second-degree sinoatrial block (Figure 1B). However, the patient had no specific symptoms other than mild palpitations and chest tightness; therefore, an atropine challenge test was not performed. Lung auscultation revealed scattered pulmonary rhonchi and mild rales in both lungs. Chest radiography revealed fibrotic tuberculous lesions without acute processes. Blood biochemical tests revealed a white blood cell count of 8.06 × 10^9^/L, polymorphonuclear neutrophils level of 96.2%, and high-sensitivity C-reactive protein level of 106.0 mg/L, indicating the presence of infection or inflammation. Other biochemical tests revealed a creatine kinase (CK) level of 109 IU/L, CK-MB level of 16 IU/L, and cardiac troponin I level of 0.02 ng/ml. In addition, serological tests for Hepatitis B, syphilis, and HIV were negative.

Given that the condition of the patient was stable, the emergency physician initially diagnosed the patient with recurrent TB or cardiovascular diseases and prescribed over-the-counter remedies, including dextromethorphan hydrobromide and traditional cough syrup, to relieve cough and reduce sputum and recommended that the patient visits the Specialty Hospital of Infectious Diseases the following day for a further comprehensive examination of the lungs and heart. Unfortunately, after returning home, the patient suddenly experienced severe chest pain, dyspnea, and cardiac arrest at midnight and died after unsuccessful resuscitation attempts. Following the request of the patient's family and the law of China, the body was delivered for a forensic autopsy to examine the cause of the unexpected death.

**Figure 2:**
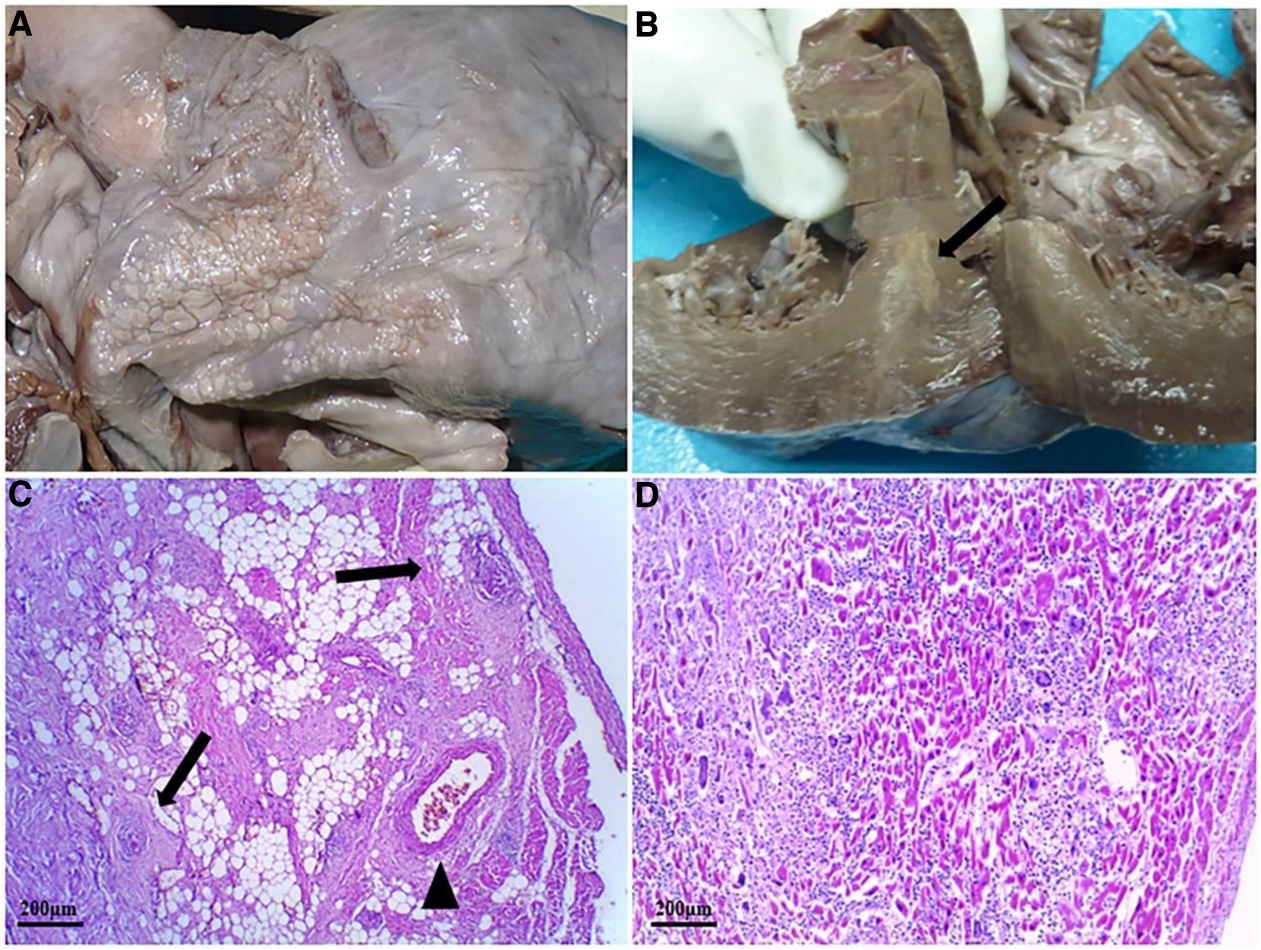
Morphological and histological examination of the heart. (**A**) Small off-white tubercles dotted the epicardium of the right auricle and right atrium. (**B**) The cut section of the myocardium of the interventricular septum and the anterior wall of the left ventricle shows whitish lesions (black arrow). (**C**) Several caseating epithelioid granulomas infiltration (black arrow) around the sinoatrial node artery (black triangle) using HE staining. (**D**) Numerous caseating necrotizing epithelioid cell granulomas in the myocardium of the left ventricle using HE staining. HE, hematoxylin–eosin staining.

**Figure 3:**
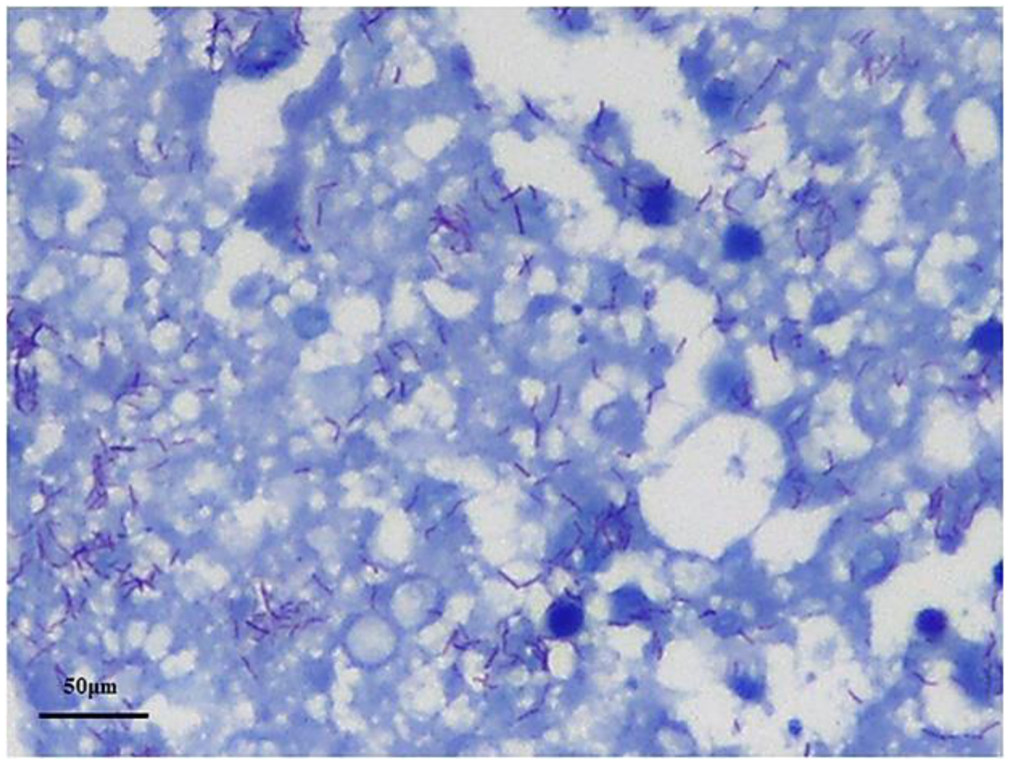
Positive Ziehl–Neelsen staining for tubercle bacilli in both lungs (oil immersion).

The autopsy revealed that the epicardium of the right auricle and right atrium were studded with slightly off-white tubercles (Figure 2A). The cut surface of the myocardium showed irregular gray-white lesions on the ventricular wall (Figure 2B). The main trunk of the left coronary artery showed the presence of atheromatous plaques with lumen stenosis (<25%). All other branches of the coronary artery were normal, and there was no evidence of pleural or pericardial involvement. Multiple white nodules, 1–2 mm in diameter, were observed with partial calcification in cut sections of the lungs. The hilar lymph nodes were grayish-white in color and enlarged. No significant abnormalities were observed in any other organ. Histological examination of the sinus node region revealed granulomas, focal fibrosis, and fatty infiltration (Figure 2C). The myocardium also showed a broad context of fibrosis with infiltrated lymphocytes, many Langhans giant cells, and caseating epithelioid granulomas (Figure 2D). Although acid-fast bacillus was not detected in the myocardium, tubercle bacillus deoxyribonucleic acid (DNA) was confirmed by real-time polymerase chain reaction (PCR) on formalin-fixed lung tissue. Tubercle bacilli were detected in the granulomatous areas of the lung tissue using Ziehl–Neelsen (ZN) staining (Figure 3).

## Discussion

TM is a rare but recognized the cause of sudden death. Currently, there are no reliable statistics regarding its incidence (12), although previous studies have reported that the frequency of myocardial TB is approximately 0.14% at autopsy, and most TB involvement is localized to the pericardium (10, 13). The relative resistance to TB infection may be related to the protective effect of myocardial motion and lactic acid production (14). Cardiac involvement is generally secondary to TB foci in other organs and is caused by hematogenous seeding, lymphatic spread, and contiguously from the pericardium (15). In the present case, TB lesions existed primarily in the lungs and myocardium but did not affect mediastinal lymph nodes or the pericardium. To our knowledge, this is the first reported case of pathologically proven TM, potentially with sinus node involvement, making this case very unusual. Histologically, TM is classified into three distinct types: miliary (due to systemic hematogenous dissemination), caseating nodular, and uncommon diffuse infiltration with lymphocytes and macrophages (16). Based on pathological examination, the present case belongs to the third category.

The clinical presentation of TM varies but is generally associated with the disease severity and affected site, ranging from asymptomatic, pericarditis, arrhythmias, valve dysfunction, ventricular outflow tract obstruction, and impaired myocardial contractility to heart failure (17). Prior to death from TM, most patients have no relevant medical history and are asymptomatic. In some cases, the initial clinical symptoms of TM are similar to those of myocardial ischemia (18). In the current case, the patient presented with myocardial involvement, including chest pain, dizziness, and amaurosis, several weeks before admission, suggesting possible myocardial insufficiency. However, there was no increase in sensitive biomarkers of myocardial injury after admission, except for an abnormal ECG of sinus node conduction. Schnitzer reported a case of miliary TM with palpitations and ventricular tachycardia as the initial manifestations, and the patient eventually died of ventricular arrhythmia (19). In contrast, Dı´az-Peromingo et al. described a cured patient with myocardial TB who was clinically characterized by long QT syndrome (20). Furthermore, Darwish et al. reported a patient with a TB infection of the whole heart who presented with atrial fibrillation and a 1:1 atrioventricular block, and sinus rhythm was restored after anti-TB treatments (21). These case studies illustrate that the presentation of patients with myocardial TB is varied and complex, and early diagnosis and treatment may prevent fatal outcomes. In the current case, in the absence of heart failure and other cardiac pathologies (including coronary and valvular diseases), the presentation of sudden dyspnea and cardiac arrest before death suggests the occurrence of severe arrhythmia, which may be directly related to the involvement of the sinus node. Symptoms of impaired cardiac function, granulomatous lesions of the sinus node, and electrocardiographic evidence of sinus node conduction abnormalities support this hypothesis of sudden death.

Diagnosis of TM remains challenging because consensus-based diagnostic criteria are lacking (18, 22). Histopathological evaluation remains the standard method for determining the presence of TB lesions. Other confirmatory examinations, including acid-fast staining and PCR, have widely been used to detect TB infection. However, *Mycobacterium TB* has been reported to yield negative results using these methods in several cases. In this study, we analyzed myocardial TB-related sudden deaths since 1970 (Table 1) and found that, of the 12 cases identified, 4 were positive for acid-fast bacilli staining, 6 were negative, and the remaining were not tested using ZN staining of myocardial tissue. Studies have shown that the PCR method has 96%–100% specificity for detecting *Mycobacterium TB* in pericardial fluid; however, some reports have shown that it has only 15%–20% sensitivity (17). du Toit-Prinsloo and Saayman described TM confirmed by histomorphology despite PCR results being negative (7). Researchers have speculated whether the reliability of PCR depends on the specificity of the primer and DNA concentration and may be related to endogenous inhibitors or fixation procedures (7, 29). Hence, a negative PCR result cannot rule out TB infection (30). In this present case, no tubercle bacilli were detected using ZN staining; however, the presence of tubercle bacillus DNA was identified using real-time PCR in formalin-fixed tissue.

**Table 1:** Cases of sudden death due to myocardial TB since 1970.

Authors	Year	Sex	Age	Diagnostic method	Extra- cardiac involvement
Behr et al. (23)	1977	M	21, 35	HE, ZN (−)	MLN
Wallis et al. (24)	1984	M	31	HE, ZN (−)	Lung, liver, kidney, MLN
Chan and Dickens et al. (13)	1992	M	71	HE, ZN	Lung, liver, spleen, kidney, bone marrow
Dada et al. (11)	2000	M	25	HE, PCR, ZN (−)	Pericarditis
Biedrzycki and Baithun (14)	2006	F	20	HE, ZN	Lung, kidney, liver
Silingardi et al. (9)	2006	F	33	HE, PCR, ZN (−)	Lung, MLN, liver, spleen
Amonkar et al. (25)	2009	F	65	HE, ZN (−)	Liver
Kanchan et al. (26)	2010	M	58	HE	None
du Toit-Prinsloo and Saayman (7)	2016	M	35	HE, ZN (−), PCR (−)	Lung, spleen
Kumar et al. (8)	2018	M	40	HE, ZN	Not examination
Chan (27)	2018	M	56	HE, ZN, Culture	Lung, diaphragm, spleen
Paliwal et al. (28)	2021	F	17	HE	Kidney, paratracheal, MLN

TB, tuberculosis; HE, hematoxylin–eosin staining; ZN, Ziehl–Neelsen staining; MLN, mediastinal lymph nodes; M, male; F, female; PCR, polymerase chain reaction; (−), negative.

A definitive diagnosis of myocardial TB can only be made from an endomyocardial biopsy (EMB) sample in clinical settings (31); however, no previous studies have examined the application of EMB sampling to cardiac TB. Recently, several studies have identified that cardiac magnetic resonance (CMR) imaging can differentially identify TM in patients with normal troponin levels and ECG findings (1). More so, positron emission tomography-computed tomography can distinguish between infiltrative myocardial TB and cardiac sarcoidosis, which could help delineate the extent of TB involvement and guide the biopsy procedure (32).

Conclusively, TM is a rare and fatal disease that is usually diagnosed postmortem due to its typically asymptomatic presentation. Without consensus on diagnostic criteria for myocardial TB, histopathological assessment remains the dominant method for identifying TB lesions. In this case, we have highlighted the co-application of acid-fast staining and PCR techniques to definitively diagnose a case of suspected TB. In future suspected cases of TB, physicians should consider the possibly enhanced risk of TM in patients with a history of TB, signs of myocardial involvement, and abnormal ECG findings.

## Data Availability

The original contributions presented in the study are included in the article, further inquiries can be directed to the corresponding author.
